# Diabetic Retinopathy and Insulin Insufficiency: Beta Cell Replacement as a Strategy to Prevent Blindness

**DOI:** 10.3389/fendo.2021.734360

**Published:** 2021-11-29

**Authors:** Eli Ipp

**Affiliations:** Department of Medicine, The Lundquist Institute at Harbor-University of California Los Angeles (UCLA) Medical Center, Torrance, CA, United States

**Keywords:** diabetes, retinopathy, beta cell (B-cell), insulin secretion, beta cell failure

## Abstract

Diabetic retinopathy (DR) is a potentially devastating complication of diabetes because it puts patients at risk of blindness. Diabetes is a common cause of blindness in the U.S. and worldwide and is dramatically increasing in global prevalence. Thus new approaches are needed to prevent this dreaded complication. There is extensive data that indicates beta cell secretory failure is a risk factor for DR, independent of its influence on glycemic control. This perspective article will provide evidence for insufficient endogenous insulin secretion as an important factor in the development of DR. The areas of evidence discussed are: (a) Presence of insulin receptors in the retina, (b) Clinical studies that show an association of beta cell insufficiency with DR, (c) Treatment with insulin in type 2 diabetes, a marker for endogenous insulin deficiency, is an independent risk factor for DR, (d) Recent clinical studies that link DR with an insulin deficient form of type 2 diabetes, and (e) Beta cell replacement studies that demonstrate endogenous insulin prevents progression of DR. The cumulative data drive our conclusion that beta cell replacement will have an important role in preventing DR and/or mitigating its severity in both type 1 diabetes and insulinopenic type 2 diabetes.

## Introduction

Diabetic retinopathy (DR) is a devastating complication of diabetes because it can lead to visual impairment and blindness. Diabetes is the most common cause of blindness in the working age population of the U.S. As prevalence of diabetes increases worldwide, new approaches are needed to prevent blindness. Pathogenesis of DR is multifactorial, but there is increasing evidence that diminished beta cell function is an independent risk factor for DR. This perspective article will put forth evidence for the role of insufficient endogenous insulin as an important factor in the development of DR that may therefore be amenable to prevention by beta cell replacement.

**Figure 1:**
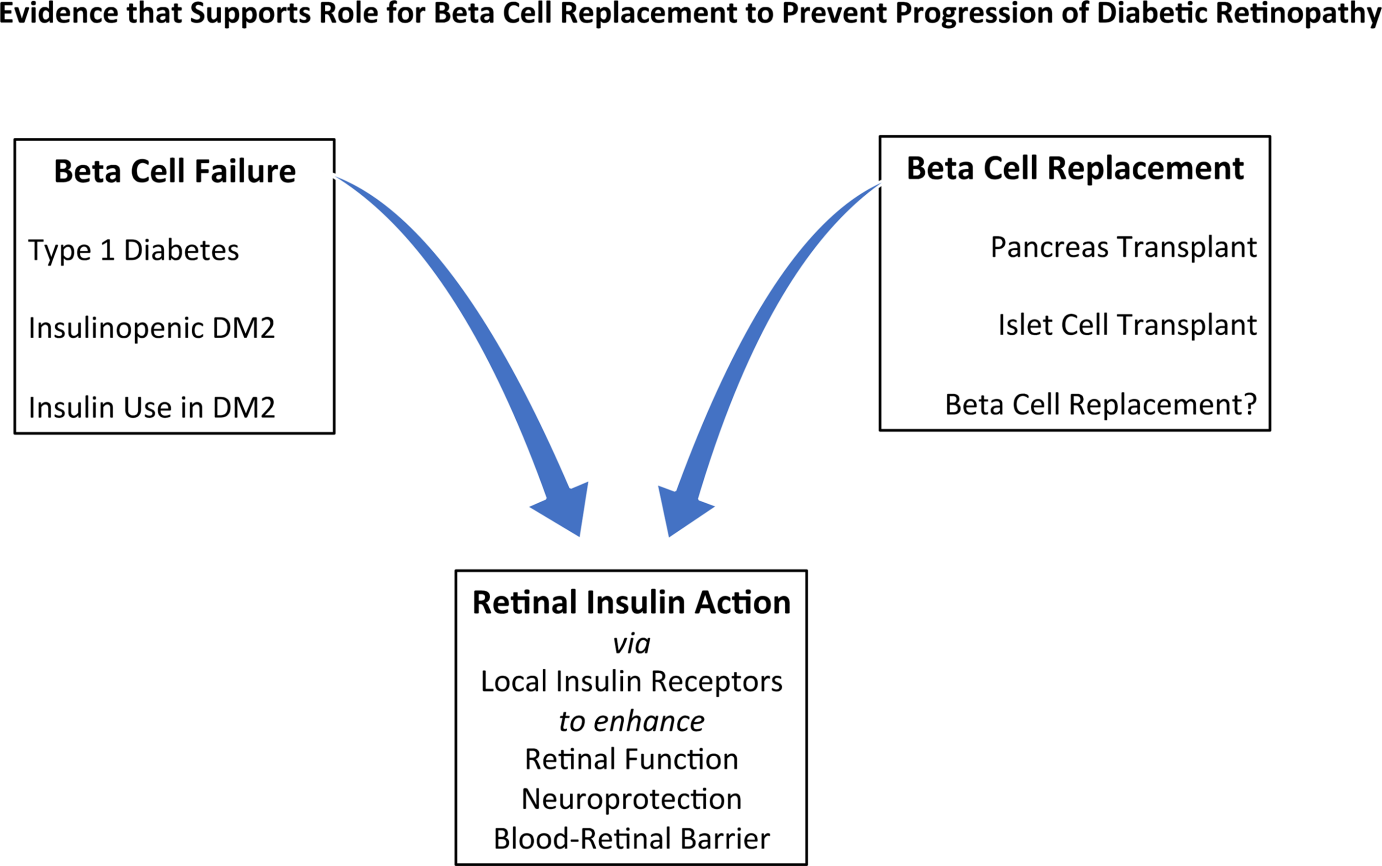
Evidence that Supports a Role for Beta Cell Replacement to Prevent Progression of Diabetic Retinopathy: insulin deficiency in the role of risk factors, effects of replacement methods, and retinal effects of insulin. DM2 = Type 2 Diabetes.

There are five areas of evidence that will be discussed (**Figure 1**):

(a) Evidence for, and role of, insulin receptors in the retina.
(b) Clinical data that demonstrate an association of beta cell insufficiency with the presence and severity of DR.
(c) Therapeutic use of insulin in type 2 diabetes as a marker for insulin deficiency and thus as an independent risk factor for DR.
(d) Recent prospective clinical studies linking development of DR with a newly described subtype of type 2 diabetes, that is characterized by insulin insufficiency.
(e) Pancreas and islet transplantation as a source of endogenous insulin and C-peptide that have a beneficial effect on DR.

The accumulation of data that derives from these disparate areas of investigation drives our conclusion that beta cell replacement will have an important role in preventing DR and/or mitigating its severity in both type 1 and insulinopenic type 2 diabetes.

### Insulin Receptors in the Retina

In order to consider a direct link of insulin secretion to DR that does not involve other biological pathways, evidence for insulin action in the retina is essential. The presence of retinal insulin receptors is thus an important pre-requisite. Though the retina is not regarded as a typical end organ for insulin action such as muscle, fat and liver - precedent exists for insulin receptors in tissues that are not classical insulin targets. The retina is indeed a non-classical target for insulin action, as evidenced by the finding that insulin receptors are widely expressed in the human retina (1). Insulin receptors have been localized to the nerve fiber layer, ganglion cells, M̈ller glia, outer nuclear layer, inner segments of rods and cones, the outer limiting membrane and retinal pigment epithelium (RPE) (1). Also, insulin signaling within the RPE occurs in dose response fashion (2).

DR has traditionally been defined by vascular changes in the retina, which form the basis for grading and classification of DR (3). It is now thought that neural involvement is an equally important participant in the pathogenesis of DR (4). It is therefore relevant that insulin has been proposed as an important neuroprotective factor (5–16), potentially protecting against the development and progression of DR. Insulin receptors are widely expressed in the neural retina (1). In rats with streptozotocin-induced diabetes, insulin has been reported to protect retinal function, reducing retinal cell apoptosis, glial activation, VEGF upregulation, and Brain-Retinal-Barrier damage (5). Insulin receptors expressed by the RPE appear to provide support to photoreceptors in the diabetic retina (6). Recent data also suggest that, even more than insulin, the pro-hormone proinsulin may exert significant neuroprotective actions in the retina (7).

In addition to the finding of receptors, insulin mediated intracellular pathways have been shown to play a role as pro-survival factors in the retina, acting through phosphoinositide-3-kinase (PI3K), protein-kinase B (Akt) and the ribosomal protein S6 kinase, p70S6K, to protect the retina from apoptosis or stress; including damage due to light (8–10). Loss of the insulin receptor in rod photoreceptors leads to diminished PI3K and Akt signaling and increased sensitivity to light induced photoreceptor degeneration (11). Insulin has also been shown to be necessary for photoreceptor survival and activity (11–15). The insulin receptor-PI3K signaling pathway provides neuroprotection to cones (16). Loss of PI3K in cones triggers cone degeneration not protected by rod-derived cone survival factors. It has therefore been suggested that the insulin receptor-PI3K signaling pathway may be a target for neuroprotective therapeutic intervention (16).

Thus, accumulating evidence supports both the presence of insulin receptors in multiple sites in the retina as well as a role for insulin mediated pathways in maintaining normal physiological function. Though more work needs to be done in this area, it appears that a basic cellular framework exists for insulin action in the retina including a likely neuroprotective role.

### Clinical Studies

Glycemic control is a key risk factor in determining the development of DR in type 1 and type 2 diabetes (17, 18), and this information has driven the adoption of glycemic goals as standards of care for diabetes management, at HbA1c levels of 7%. Though key to the development and progression of DR, the mechanisms by which glycemia influences the development of DR have been questioned (19). Also, glycemic control as a management strategy may have its limitations in DR prevention, as shown in studies of type 2 diabetes in which further improvement in HbA1c below 7% did not provide additional benefit to reduce DR (20, 21). DR progressed in these studies, independent of improved glycemic control. This strongly suggests that factors other than glycemic control may be involved in the progression of DR.

In type 1 diabetes, residual endogenous insulin secretion was first shown to protect patients against development of DR (22). In an early follow-up (6 years) of the Diabetes Control and Complications Trial (DCCT) beta cell function, measured as residual C-peptide, showed a clear protective role in DR. At that stage of the trial, there was a sufficient number of participants with some residual C-peptide secretion (56%) to demonstrate this protective effect (22). But there are limits to this protection. In long-standing type 1 diabetes when beta cell function is so depleted, protection against DR no longer occurs (23). In the latter report, after 37 years of DCCT and its follow up study (DCCT/EDIC), only 12% had residual C-peptide responses, providing an explanation for the lack of a demonstrated beneficial effect on DR.

Importantly, in type 1 diabetes glycemic control was shown to be irrelevant to protection provided by endogenous beta cell secretion – in the 6 year DCCT trial, an effect of residual C-peptide to protect the retina was found, irrespective of the A1C achieved in each of the experimental arms (22). In other words, even in the ‘protected’ group in good glycemic control of A1C about 7%, the development of DR was significantly dependent on residual C-peptide. This suggests that in addition to the effects of glycemic control, other factors are also relevant – specifically, residual beta cell function.

In type 2 diabetes, a clear relationship between residual insulin and DR was initially more difficult to show. Some studies demonstrated that lower levels of C peptide are associated with DR (24–27), while other studies did not (28–30). The GOLDR (Genetics of Latino Diabetic Retinopathy) cohort (31) was an opportunity to resolve this question by introducing three distinct methodological advantages: a cohort large enough to include analyzable subgroups, measurement of circulating insulin in addition to C-peptide, and statistical approaches to deal with specific confounders. This study confirmed an inverse correlation of DR with C peptide and insulin in type 2 diabetes (31).

The GOLDR study (31) differed from previous studies in that it examined the relationship of C-peptide and insulin within the entire spectrum of DR severity and used 7-field digital imaging with standard ETDRS grading criteria. This was the first study to measure insulin as well as C-peptide concentrations, to better represent residual beta-cell function. It was also the first study to evaluate all categories of DR severity and residual beta cell function. The deficit in endogenous insulin secretion was associated with increasingly severe DR. Plasma C peptide concentrations paralleled plasma insulin, suggesting that insulin concentrations reflect insulin secretion— and therefore beta-cell function. This association of circulating insulin and C peptide with the presence and severity of DR remained highly significant after adjusting for well-known risk factors for DR, including diabetes duration and notably, was independent of glycemic control (31). A concurring earlier study found lower post-prandial insulin associated with DR (32).

Insulin secretory failure is a complex outcome to evaluate in clinical studies of diabetic complications, such as DR. Circulating insulin reflects rates of secretion and clearance. Renal clearance is the main mechanism for C peptide degradation, while insulin is also cleared by the liver. The more severe DR, the more likely renal clearance will be impaired and therefore that insulin and C-peptide concentrations will be increased, no longer reflecting accurately rate of insulin secretion. By appropriate statistical analysis, Kuo et al. confirmed that worse DR occurred with decreasing C peptide, reinforcing the importance of deficient beta-cell function in DR (31).

It is therefore likely that discrepant results in earlier type 2 diabetes studies, all of which reported C peptide/DR relationships without insulin measurements, may be due to the complexity of using C peptide as a marker of beta-cell function in the presence of kidney disease.

The results of the GOLDR study suggest that besides attention to glycemic control, maintenance of beta-cell function or beta-cell mass in type 2 diabetes may also be justified as a therapeutic goal, thus warranting further investigation (31).

### Therapeutic Insulin Use as a Risk Factor for DR in Type 2 Diabetes

Multiple studies of type 2 diabetes have shown that use of insulin to manage glycemic control is an independent risk factor for DR (33–35). Tudor (33) in the San Luis Valley Diabetes Study, Colorado found an odds ratio for any DR (95% CI) = 8.45 (2.65-26.97), in patients on insulin *vs.* no medications. Jones (34) in the Norfolk Diabetic Retinopathy Study showed that compared to diet control only, the adjusted hazard ratio (95% CI) = 2.17 (1.68–2.81) if participants were using insulin. Thomas (35) compared insulin use to diet control with an adjusted hazard ratio = 2.03 (1.89–2.18) for DR.

What could account for this relationship between use of insulin and the increased likelihood of developing DR? The reflex conclusion has been that insulin use signals poor glycemic control, and while this is generally true, it has led to overlooking the possibility that insulin use reflects beta cell failure and that lack of endogenous insulin might have a direct influence in DR pathogenesis. Impairment of beta cell function is integral to the pathogenesis of type 2 diabetes, but most patients nevertheless do not require insulin treatment to control blood glucose. Only those with more severe beta cell decompensation require exogenous insulin to manage their diabetes, and so insulin treatment becomes a de facto marker for endogenous insulin deficiency in the face of the insulin resistance in type 2 diabetes (36).

Modern strategies to prevent diabetic blindness focus on risk factors that are amenable to change, such as glycemic and hypertension control (37). Future approaches to prevent blindness will also use large data to derive DR risk stratification protocols to identify specific patients at high risk to target for risk factor amelioration. Insulin use per se is not usually targeted because the assumption is that it is a marker for poor glycemic control rather than a marker for endogenous insulin deficiency in the pathogenesis of DR. This perspective article argues for endogenous insulin deficiency to be recognized in its own right as a direct risk factor independent of glucose control. In this setting, replacement using beta cells can be seen as a logical strategy once methods are developed that simplify the methodology and make it available for more widespread use, such as immune evasion and use of insulin secreting islet organoids (38).

### New Prospective Clinical Studies Identifying an Insulinopenic Sub-Type in Type 2 Diabetes

Type 2 diabetes has long been considered a heterogeneous disease (36). The classical phenotype of an obese middle-aged person with insulin resistance, hypertension, dyslipidemia and increased waist circumference does not include all patients with type 2 diabetes, some of whom are older, leaner, or more insulin sensitive (39). In 2018, Ahlqvist introduced a novel approach to classification of diabetes using cluster analysis. These investigators identified four different clusters of type 2 diabetes, with an additional autoimmune cluster defined by positive anti-GAD antibodies (39). Most notably, a type 2 cluster identified as an insulin deficient group was also the most likely cluster to develop DR. Thus, this clinical study demonstrated rather conclusively in prospective fashion that belonging to an endogenous insulin deficient group was a risk factor for DR. Another (insulin resistant) cluster were more likely to develop diabetic kidney disease.

These type 2 diabetes clusters, defined at diabetes onset seem to remain consistent even after several years of diabetes, suggesting that the degree of homogeneity within clusters might reflect different etiologies or pathogeneses that remained true despite the passage of time (40). These clusters were confirmed in other populations in China (41) and the U.S. (41). In a retrospective Japanese study, cluster analysis revealed an association between an insulin deficient cluster and DR (42). In Asian Indians, a cross-sectional study of about 20,000 patients with type 2 diabetes showed a similar relationship with DR and the insulin deficient cluster (43).

Of importance for the view presented in this perspective, is the identification of the insulin deficient cluster, and that it was associated with a higher incidence of DR (40, 42, 43). This provides powerful new information illustrating that in type 2 diabetes, endogenous insulin and/or C-peptide (44) secretion, when it fails, may be responsible for development or progression of DR.

In another large prospective trial (Veterans Affairs Diabetes Trial - VADT) of the effect of tight glycemic control on DR, no benefit was found with better A1C in the intervention arm (45). Despite this, in the entire cohort higher baseline C-peptide concentrations were protective for both incidence and progression of DR (45). DR incidence was reduced by 67.2%, and DR progression was reduced 47% for each 1 pmol/ml increase in baseline C-peptide, respectively, another indication that residual insulin secretion may influence DR independent of glycemic control.

### Results of Pancreas and Islet Transplantation as a Source of Endogenous Insulin and C-Peptide on DR

If endogenous insulin deficiency contributes to DR, can replacement of beta cells reverse this course? If endogenous insulin is more effective in preventing DR progression than the exogenous form, a positive effect upon DR after beta cell replacement should be found. Indeed, data derived from clinical transplant studies in type 1 diabetes support a protective role in DR for endogenous insulin secretion.

In a recent meta-analysis (46) islet cell transplantation was associated with diminished DR progression compared with either intensive or standard medical therapy (5.2% *vs* 25.0%; relative risk=0.25; 95% CI, 0.08–0.71). This meta-analysis included a total of 193 type 1 diabetic patients in 3 different studies, with duration of diabetes in excess of 20 years, and follow-up after transplant for a median of 5-7 years (46, 47).

Data from pancreas transplants compared with non-transplanted diabetic controls shows similar evidence (46). Isolated pancreas transplantation (RR=0.16; 95% CI, 0.05–0.49) and combined pancreas-kidney transplantation (RR 0.21; 95% CI, 0.09–0.52) were associated with reduced risk of DR progression compared with controls. (46, 48). These studies were carried out in 159 patients in total, but with a shorter median follow up, only 18-29 months. Mean diabetes duration was also greater than 20 years prior to transplant.

These beneficial effects after transplant cannot prove, but are consistent with, a role for beta cell secretion products over exogenous insulin in preventing or reversing progression of DR. More data are needed to demonstrate a firm role for endogenous insulin that also take into account the possible influence of other factors, such as glycemia, islet isolation methods, and immunosuppressive regimens. Notwithstanding these factors, the transplant data also support a role for endogenous insulin in DR protection.

There are, however, two important caveats that need to be mentioned: (a) results of short-term follow up studies of pancreas transplant and DR, and (b) a putative role for C-peptide in DR.

The early period after pancreas transplantation is a complex setting to assess the effect of beta cell replacement because two dominant processes occur simultaneously: successful re-initiation of beta cell function and endogenous insulin secretion; and rapid decline in glycemia. These two processes have opposite effects on the retina. In the case of rapid glucose control, worsening is a well-documented phenomenon (49, 50). After pancreas transplant, similar deterioration has been demonstrated in short-term trials, conceivably explaining DR progression within a few months of transplant (51, 52), as opposed to the longer follow-up trials mentioned above (46–48). It is therefore essential to examine the effect of pancreas transplant early and also after long-term follow-up.

Since endogenous beta cell function consists of both insulin and C-peptide secretion, it should be noted that there are data that support the concept that there is a direct biological effect of C-peptide on the microvasculature (44). C-peptide has been shown to decrease glucose-induced apoptosis of endothelial cells, prevent oxidative stress through an antioxidant role, reducing RAC-1 translocation to the membrane and NAD(P)H oxidase activation (53). Thus it is conceivable that the role described here for residual endogenous beta cell secretion may be mediated by C-peptide action as well as, or instead of, insulin.

## Discussion and Conclusions

In this perspective, we have assembled information available to support the concept that endogenous insulin secretion plays an important role in the prevention of DR, that is not achieved by exogenous insulin alone. The presence of insulin receptors in the retina sets the stage for an active biological role for insulin in the eye. Although exogenous insulin may also interact with these insulin receptors, evidence is presented that supports a role for endogenous insulin in protecting against DR in both type 1 and type 2 diabetes. Insulin treatment in type 2 diabetes, reflecting endogenous insulin insufficiency, is an important risk factor for DR. Cross-sectional and prospective clinical studies accentuate the role of endogenous insulin deficiency in the development of DR in both type 1 and type 2 diabetes. Replacing beta cells by islet cell transplants prevents progression of DR in people with type 1 diabetes, direct support for the importance of endogenous insulin secretion to prevent DR. A caveat for many of these studies is that insulin deficiency is commonly associated with poor glycemic control, which is itself a risk factor for DR. However many of the studies quoted in this perspective either adjusted for glycemic control or demonstrated a C-peptide benefit even in tightly controlled patients; thus insulin deficiency remained an independent risk factor for DR.

The accumulation of data presented here strongly suggests that when beta cell replacement is more readily available, an additional indication for its use could be to protect the retina in patients with type 1 diabetes and even insulin deficient type 2 diabetes, an even larger population who could potentially benefit from this approach.

Future prospective clinical studies of beta cell replacement should include progression of DR as an essential outcome measure.

## Data Availability Statement

The original contributions presented in the study are included in the article/supplementary material. Further inquiries can be directed to the corresponding author.

## Author Contributions

The author confirms being the sole contributor of this work and has approved it for publication.

## Conflict of Interest

The author declares that the research was conducted in the absence of any commercial or financial relationships that could be construed as a potential conflict of interest.

## Publisher’s Note

All claims expressed in this article are solely those of the authors and do not necessarily represent those of their affiliated organizations, or those of the publisher, the editors and the reviewers. Any product that may be evaluated in this article, or claim that may be made by its manufacturer, is not guaranteed or endorsed by the publisher.
